# Conditioning factors for the scientific productivity of undergraduate students of health sciences at a private Peruvian University: A cross-sectional analytical study

**DOI:** 10.12688/f1000research.143021.1

**Published:** 2024-01-03

**Authors:** César Antonio Bonilla-Asalde, Isabel Cristina Rivera-Lozada, Oriana Rivera-Lozada

**Affiliations:** 1 Escuela profesional de salud Humana, Universidad Privada San Juan Bautista, Lima, Peru; 2 Departamento de economía, Universidad del Cauca, Popayán, Colombia; 3 Facultad de educación, Universidad Nacional Mayor de San Marcos, Lima, Peru

**Keywords:** Higher education, scientific research, student research, research universities

## Abstract

**Background:**

The objective of this study was to determine the conditioning factors for scientific research productivity in university students of health sciences.

**Methods:**

A cross-sectional analytical observational study was conducted. The study population was 4104 students enrolled during the 2021-I semester in the Faculty of Health Sciences of a private Peruvian university. A sample size of 400 students was determined and a stratified probability sampling was used. The variables were measured through surveys. The dependent variable was scientific research productivity, and the independent variables were institutional culture, knowledge management and technological capital. Summary measures are reported according to the type of variable. The chi-square test with a significance level of p<0.05 was applied to assess the association between the variables of interest. A multiple logistic regression analysis was performed using the Stepwise method. Prevalence ratios (PR) with their respective 95% confidence intervals (95%CI) were calculated.

**Results:**

From the total of 400 students, 74.5% were male, 57.25% were aged between 18 and 27 years, 17% belonged to the school of human medicine and 72% were in their sixth year of studies. Scientific research productivity was associated with management commitment (PR=1.493; 95%CI: 1.077–2.068, p=0.015), sense of personal growth (PR=1.632; 95%CI: 1.041–2.558; p=0.024), recognition by the university (PR=1.385; 95%CI: 1.012–1.896; p=0.043), strategic alliances (PR=1.422; 95%CI: 1.032–1.959; p=0. 03), having research proposals (PR=1.522; 95%CI: 1.114–2.08; p=0.009), dissemination of results obtained (PR=1.542; 95%CI: 1.12–2.122; p=0.01), availability of human resources (PR=1.591; 95%CI: 1.165–2.173; p=0.004), access to equipment and software (PR=1.482; 95%CI: 1.061–2.069; p=0.018) and to laboratories (PR=1.438; 95%CI: 1.047–1.974; p=0.024).

**Conclusion:**

It is concluded that the research productivity of undergraduate students of health sciences is low. It is necessary to strengthen research promotion measures in universities for the control and monitoring of students’ scientific participation.

## Introduction

Nowadays, the academic development of universities is oriented primarily to the training of human talent, research and technological progress, a situation that is reflected in the research indicators used to determine the level of competitiveness, academic quality and resource allocation. In the face of this reality, university directives increase the demands on teachers, students and researchers in order to raise academic standards as well as the position in the various rankings that serve as a comparative benchmark in the educational system ( Sirvent *et al.*, 2016).

It is interesting to mention that, unlike countries in Europe, Asia and Oceania, in Latin America three quarters of research is concentrated in universities and mainly in public ones, tipping the balance towards conducting applied research rather than basic research, which is oriented towards the development of science and technology ( Alarco, Changllio-Calle and Cahuana-Salazar, 2017).

Research published in journals of importance in the region of the Americas exceeds 100,000 titles in the period 1996–2003; however, very few correspond to production by university students, 75% belong to Brazil, Argentina, Chile and Mexico, placing Peru in 8th place with 3% in that concept ( Osada, Loyola-Sosa and Berrocal, 2014).

In Peru, research in university campuses is limited by a myriad of factors, including state investment, the lowest in this hemisphere -0.1% of GDP, below Brazil, Mexico, Colombia and Chile. This information allows us to understand why in the period prior to the enactment of the 2014 University Law, only three universities, one national and two private, concentrated 64% of the scientific production ( Perdomo *et al.*, 2020).

Moquillaza (2019) draws attention to the scarcity of information in indexed journals related to the scientific production of students in higher education institutions and the small number of journals that accept research conducted by students. Another aspect to point out in the Peruvian case has to do with the limited number of journals corresponding to university publishers that do not exceed 10% of these publications.

While as from 2018 there is evidence of greater research in Peru, it remains globally low; among the possible causes is the lack of indexed journals for research authored by students. Frequencies of reports are found in Colombia 11%, Chile 10% and similar in Peru. Student perception of the causes, besides the above, are related to the lack of research experience of the teaching staff and that the contents provided in research methodology courses are insufficient, in addition to not teaching or giving guidelines on writing an article and how to submit it to a journal for its publication ( Santibáñez, 2017; Castro-Rodríguez, 2019).

Castro-Rodríguez *et al.* (2018), explain that only 4.5% of papers published in Scielo Peru had a contribution from at least one student from a Peruvian university, reflecting the pitfalls faced by university students when publishing an academic paper, as well as the lack of opportunities to be incorporated into research groups, a deficit in incentives and the underestimation of student capacities to develop quality research. Therefore, the objective of this research was to determine the conditioning factors for the scientific research productivity of undergraduate students of health sciences at a private university in Peru.

## Methods

### Study design and population

An analytical cross-sectional observational study was conducted. The study population consisted of 4104 undergraduate students. The study included students of both genders enrolled in the 2020-I semester at the Faculty of Health Sciences of the Norbert Wiener Private University and who had taken research subjects. Students who did not wish to participate in the study and those who did not complete the requested information properly were excluded.

### Type of sampling and sample size

A sample size of 400 students was calculated and a stratified probability sample was made by proportional allocation among the students who met the selection criteria of the study.

### Study variables

The independent variables included institutional culture, knowledge management and technological capital measured on a nominal scale. The dependent variable was scientific research productivity.

### Procedures

The information from the participants was gathered through four questionnaires ( Bonilla-Asalde *et al.,* 2023b) based on the model of Rueda-Barrios and Rodenes-Adam (2016). All questions were posed according to a dichotomous qualitative scale (1=Yes, 2=No). Three questionnaires were applied for the independent variables. The first was composed of 24 questions on institutional culture and included participatory culture, motivational culture and teamwork culture as dimensions. The second questionnaire was composed of 13 questions on knowledge management and included the dimensions of socialization, externalization and internalization. The third questionnaire was composed of 15 questions on technological capital and included the dimensions R&D investment, technological endowment and technological tools. For the dependent variable, a seven-question questionnaire was applied with the dimensions of publications and visibility of the researcher. Data gathering was conducted via email during the period of March–August 2021.

The data gathering instruments were developed by the research team and validated by Aiken’s V coefficient, and the binomial test with the participation of ten methodological and thematic experts in university education who assessed the clarity, objectivity, updating, organization, sufficiency, adequacy, coherence, methodology and relevance of the content of the instruments. Reliability was assessed through a pilot test that included 30 students from the Faculty of Health Sciences of the Norbert Wiener Private University. The four instruments were subject to the Kuder Richardson test (KR-20) and a KR coefficient equal to 0.80 was obtained for the questionnaire on institutional culture; 0.82 for knowledge management; 0.79 for technological capital and 0.86 for scientific research productivity.

### Data processing and analysis

The data obtained ( Bonilla-Asalde *et al.,* 2023a) were gathered in the Microsoft Excel 2010 software and were analyzed with the SPSS software version 25.0. Tables with absolute and relative frequencies were reported for categorical variables, while measures of central tendency and dispersion were calculated for quantitative variables.

To evaluate the relationship between each independent variable with the dependent variable, a bivariate analysis was performed using Pearson’s chi-square test with their respective p-values; values of p<0.05 were considered statistically significant. The prevalence ratio (PR) and its respective 95% confidence interval were calculated for each relationship.

Likewise, to evaluate the relationship between the independent variables and performance in scientific research, a multiple logistic regression analysis was applied, using the Stepwise method where the indicators that showed a value of p<0.05 in the previous bivariate analysis were added.

### Ethical aspects

This study was carried out following the guidelines of the 1964 Helsinki Declaration and its subsequent amendments. This study was evaluated and approved by the Institutional Research Ethics Committee of the Norbert Wiener University on February 5, 2021, expedient 521-2021. All the participants in the study signed the informed consent form before their participation and their identity was anonymized for the elaboration of the database, so their integrity was not violated.

## Results

### General characteristics of the study sample

A total of 400 students from the Faculty of Health Sciences of the Norbert Wiener Private University were analyzed, of which 74.5% (n=298) were male and 57.25% (n=229) were between 18 and 27 years of age. Also, 17% (n=68) were studying in the School of Human Medicine, while 5% (n=20) in the School of Human Nutrition. Besides, 78% (n=288) were in their sixth year of studies, while 0.5% (n=2) were in their eleventh year ( Table 1).

**Table 1.** General characteristics of the students of the Faculty of Health Sciences of the Norbert Wiener Private University, Peru 2021 (n=400).

Characteristics	n	%
**Gender**		
Male	298	74.5
Female	102	25.5
**Age (years)**		
18-27	229	57.25
28-37	137	34.25
38-47	15	3.75
48 or more	19	4.75
**Civil status**		
Single	347	86.75
Married	33	8.25
Divorced/separated	1	0.25
Widowed	4	1.00
Cohabitant	15	3.75
**School of the participant**		
Human Medicine	68	17.00
Obstetrics	32	8.00
Psychology	32	8.00
Medical technology – physical therapy and rehabilitation	36	9.00
Medical technology – clinical laboratory and anatomical pathology	32	8.00
Nursing	64	16.00
Dentistry	51	12.75
Human Nutrition	20	5.00
Pharmacy and Biochemistry	65	16.25
**Semester of studies**		
Sixth	288	72.00
Seventh	47	11.75
Eighth	29	7.25
Ninth	5	1.25
Tenth	29	7.25
Eleventh	2	0.5
Twelfth	0	0.00

### Descriptive analysis of the independent and dependent variables in the study sample

The indicators of institutional culture were evaluated in the sample studied. 70.75% (n=283) stated that there are no agreements for the dissemination of research. 76% (n=304) expressed the opinion that there is a concern for the personal growth of students, 65.5% (n=262) expressed interest in research and 60.25% (n=241) considered that there is no recognition of students who practice research by the university.

When evaluating the knowledge management indicators, it was found that 69.25% (n=277) consider that access to information is adequate. 77% (n=308) consider that there is adequate advice from teachers on research, while 72.5% (n=290) reported that there are no mechanisms for the dissemination of results. On the other hand, 62.75% (n=251) stated that they are not part of any scientific network and 82.75% (n=331) do not have access to indexed scientific journals. In addition, 60.25% (n=241) considered that the university does not use the results of research conducted by students.

Technological capital indicators were evaluated. It was found that 66% (n=264) consider that they do not have supporting human resources for research. 62.25% (n=249) stated that they had sufficient databases to conduct research, while 80% (n=320) considered that they did not have collaborative tools for research ( Table 2).

**Table 2.** Descriptive analysis of the institutional culture, knowledge management and technological capital of the Faculty of Health Sciences of the Norbert Wiener Private University, Peru 2021 (n=400).

Characteristics	Yes	No
	n (%)	n (%)
**Institutional culture:**		
**Participatory culture**		
Management commitment	202 (50.5)	198 (49.5)
Agreements	117 (29.25)	283 (70.75)
Research policies	207 (51.75)	193 (48.25)
**Motivational culture**		
Personal growth	304 (76.0)	96 (24.0)
Interest in research	262 (65.5)	138 (34.5)
Recognition	159 (39.75)	241 (60.25)
**Teamwork culture**		
Multidisciplinary research teams	209 (52.25)	191 (47.75)
Strategic alliances	192 (48.0)	208 (52.0)
**Knowledge management:**		
**Socialization**		
Access to information	277 (69.25)	123 (30.75)
Dissemination of results	110 (27.5)	290 (72.5)
Research proposals	140 (35.0)	260 (65.0)
Mentoring by teachers	308 (77.0)	92 (23.0)
**Externalization**		
Shared results	104 (26.0)	296 (74.0)
Participation in scientific networks	149 (37.25)	251 (62.75)
Access to scientific journals	69 (17.25)	331 (82.75)
**Internalization**		
Incorporation of lessons learned	184 (46.0)	216 (54.0)
Use of research results	159 (39.75)	241 (60.25)
**Technological capital:**		
**R&D investment**		
Availability of supporting human resources	136 (34.0)	264 (66.0)
Visibility of investment	170 (42.5)	230 (57.5)
**Technological resources**		
Equipment and software	218 (54.5)	182 (45.5)
Laboratories	180 (45.0)	220 (55.0)
**ICT tools**		
Databases	249 (62.25)	151 (37.75)
Collaborative tools	80 (20.0)	320 (80.0)

Table 3 shows the descriptive analysis of the dependent variable. It was found that 72.25% (n=289) have not published any article in indexed journals or congresses and 98.5% (n=394) do not have any research project approved for execution. Furthermore, none of the students in the study sample has an H index and only 2.5% (n=10) have at least one publication in Open Access ( Table 3).

**Table 3.** Descriptive analysis of scientific research productivity in students of the Faculty of Health Sciences of Norbert Wiener Private University, Peru 2021 (n=400).

Characteristics	Yes	No
	n (%)	n (%)
**Publications**		
Publications in indexed journals and conferences	111 (27.75)	289 (72.25)
Research projects approved	6 (1.5)	394 (98.5)
**Visibility of the researcher**		
H index result	0 (0.0)	400 (100.0)
Studies in Open Access	10 (2.5)	390 (97.5)

### Bivariate analysis of the independent variables and the dependent variable in the study sample

In the bivariate analysis, a statistically significant association was found between scientific research productivity and management commitment (CPR=1.493; 95%CI: 1.077–2.068; p=0.015), personal growth (PR=1.632; 95%CI: 1.041–2.558; p=0.024), receiving recognition for practicing research (PR=1.385; 95%CI: 1.012–1.896; p=0.043) and strategic alliances (CPR=1.422; 95%CI: 1.032–1.959; p=0.03). Regarding knowledge management, research proposals (CPR=1.522; 95%CI: 1.114–2.080; p=0.009), shared results (CPR=1.542; 95%CI: 1.120–2.122; p=0.01) and use of research results (CPR=1.436; 95%CI: 1.049–1.965; p=0.024) were significantly associated with scientific research productivity. In the technological capital dimension, the availability of human resources support (CPR=1.591; 95%CI: 1.165–2.173; p=0. 004), visibility of investment in research (CPR=1.481; 95%CI: 1.08–2.029; p=0.014), having equipment and software (CPR=1.482; 95%CI: 1.061–2.069; p=0.018) and laboratories (CPR=1.438; 95%CI: 1.047–1.974; p=0.024) ( Table 4).

**Table 4.** Bivariate analysis of the institutional culture, knowledge management and technological capital according to scientific research productivity in students of the Faculty of Health Sciences of the Norbert Wiener Private University, Peru 2021 (n=400).

Characteristics		Scientific research productivity		CPR (95%CI) *	P-value
		No (n=289)	Yes (n=111)		
		n (%)	n (%)		
**Institutional culture:**					
**Participatory culture**	Management commitment				
	Yes	135 (66.83)	67 (33.17)	1.493 (1.077-2.068)	**0.015**
	No	154 (77.78)	44 (22.22)		
	Agreements				
	Yes	82 (70.09)	35 (29.91)	1.114 (0.795-1.561)	0.534
	No	207 (73.14)	76 (26.86)		
	Research policies				
	Yes	147 (71.01)	60 (28.99)	1.097 (0.798-1.507)	0.568
	No	142 (73.58)	51 (26.42)		
**Motivational culture**	Personal growth				
	Yes	211 (69.41)	93 (30.59)	1.632 (1.041-2.558)	**0.024**
	No	78 (81.25)	18 (21.74)		
	Interest in research				
	Yes	181 (69.08)	81 (30.92)	1.422 (0.988-2.048)	0.051
	No	108 (78.26)	30 (21.74)		
	Recognition				
	Yes	106 (66.67)	53 (33.33)	1.385 (1.012-1.896)	**0.043**
	No	183 (75.93)	58 (24.07)		
**Teamwork culture**	Multidisciplinary research teams				
	Yes	146 (69.86)	63 (30.14)	1.199 (0.871-1.652)	0.263
	No	143 (74.87)	48 (25.13)		
	Strategic alliances				
	Yes	129 (67.19)	63 (32.81)	1.422 (1.032-1.959)	**0.03**
	No	160 (76.92)	48 (23.08)		
**Knowledge management:**					
**Socialization**	Access to information				
	Yes	201 (72.56)	76 (27.44)	0.964 (0.687-1.3549)	0.834
	No	88 (71.54)	35 (28.46)		
	Dissemination of results				
	Yes	75 (68.18)	35 (31.82)	1.214 (0.869-1.697)	0.263
	No	214 (73.79)	76 (26.21)		
	Research proposals				
	Yes	90 (64.29)	50 (35.71)	1.522 (1.114-2.080)	**0.009**
	No	199 (76.54)	61 (23.46)		
	Mentoring by teachers				
	Yes	224 (72.73)	84 (27.27)	0.929 (0.645-1.340)	0.696
	No	65 (70.65)	27 (29.35)		
**Externalization**	Shared results				
	Yes	65 (62.5)	39 (37.5)	1.542 (1.120-2.122)	**0.01**
	No	224 (75.68)	72 (24.32)		
	Participation in scientific networks				
	Yes	110 (73.83)	39 (26.17)	0.912 (0.654-1.273)	0.588
	No	179 (71.31)	72 (28.69)		
	Access to scientific journals				
	Yes	46 (66.67)	23 (33.33)	1.254 (0.859-1.831)	0.255
	No	243 (73.41)	88 (26.59)		
**Internalization**	Incorporation of lessons learned				
	Yes	126 (68.48)	58 (31.52)	1.285 (0.936-1.763)	0.12
	No	163 (75.46)	53 (24.54)		
	Use of research results				
	Yes	105 (66.04)	54 (33.96)	1.436 (1.049-1.965)	**0.024**
	No	184 (76.35)	57 (23.65)		
**Technological capital:**					
**R&D investment**	Availability of supporting human resources				
	Yes	86 (63.24)	50 (36.76)	1.591 (1.165-2.173)	**0.004**
	No	203 (76.89)	61 (23.11)		
	Visibility of investment				
	Yes	112 (65.88)	58 (34.12)	1.481 (1.08-2.029)	**0.014**
	No	177 (76.96)	53 (23.04)		
**Technological resources**	Equipment and software				
	Yes	147 (67.43)	71 (32.57)	1.482 (1.061-2.069)	**0.018**
	No	142 (78.02)	40 (21.98)		
	Laboratories				
	Yes	120 (66.67)	60 (33.33)	1.438 (1.047-1.974)	**0.024**
	No	169 (76.82)	51 (23.18)		
**ICT tools**	Databases				
	Yes	183 (73.49)	66 (26.51)	0.889 (0.646-1.225)	0.476
	No	106 (70.2)	45 (29.8)		
	Collaborative tools				
	Yes	52 (65.0)	28 (35.0)	1.349 (0.95-1.917)	0.105
	No	237 (74.06)	83 (25.94)		

* Crude prevalence ratio.

### Multiple logistic regression analysis according to scientific research productivity in the study sample

The multiple logistic regression model found association between scientific research performance with management commitment (APR=1.392; 95%CI: 1.001–1.935; p=0.049) and availability of human resources to support research (APR=1.471; 95%CI: 1.063–2.063; p=0.02) ( Table 5).

**Table 5.** Multiple logistic regression analysis of the independent variables according to scientific research productivity in students of the Faculty of Health Sciences of the Norbert Wiener Private University, Peru 2021.

Characteristics	APR *	95% confidence interval	P-value ^+^
Management commitment	1.392	1.001–1.935	**0.049**
Personal growth	1.492	0.945–2.354	0.086
Recognition	1.206	0.84–1.733	0.31
Strategic alliances	1.283	0.886–1.859	0.187
Research proposals	1.327	0.916–1.922	0.134
Shared results	1.314	0.867–1.992	0.198
Use of research results	1.05	0.673–1.638	0.83
Availability of supporting human resources	1.471	1.063–2.036	**0.02**
Visibility of investment	1.315	0.939–1.842	0.111
Equipment and software	1.308	0.914–1.871	0.142
Laboratories	1.278	0.919–1.778	0.145

* Adjusted prevalence ratio.

^**+**^ p-value estimated by multiple logistic regression using the Stepwise method, with significance level of p<0.05.

## Discussion

Determining the conditioning factors of scientific research productivity of undergraduate students of health sciences in order to provide relevant information that allows to know and understand the scientific behavior of this group of interest contributes to identify the university institutional responsibility as knowledge generating organizations ( Daher, Panuncio and Hernandez, 2018) as well as to account for the level of implementation of the scientific method by the student body and the research practices that manage to permeate teachers ( Chachaima-Mar, Fernández-Guzmán and Atamari-Anahui, 2019).

In contemporary society, universities, through the fulfillment of their mission of education, research and social interaction, play a transcendental role in the production and management of knowledge, innovation and technological development, for which they need to have clear, timely and relevant information regarding the factors that limit or promote scientific activity in students ( Santibáñez, 2017; López, 2019).

This study found that only 27.75% of the students included in the population have publications in indexed journals and/or congresses, and only 1.6% have approved research projects. Besides, none of the students in the sample has a registered H-index. These results evidence the low participation of students in research, as previously reported by Castro-Rodríguez (2019) with a student scientific production of 10%, different from the previous study by Castro-Rodríguez *et al.* (2018) who reported 3.5% of students with at least one publication. This low student participation in research can be explained because 72% of the sample was in the sixth cycle of studies and research courses are oriented in the last cycles of the degree.

However, we found that 76% consider it important to do research as part of their personal growth; also, 65.5% of the sample has interest in participating in research, which is far from what was reported by Rojas and Méndez (2016) who analyzed the barriers in the process of teaching research in undergraduate, showing that there is less and less interest in engaging in research, but this situation is a consequence of a decreasing trend in the attitude towards research at a higher university level and the teaching responsibility to awaken and encourage a different attitude towards research processes ( Alarco, Changllio-Calle and Cahuana-Salazar, 2017). This discordance between the high percentage of students interested in research and the low level of participation could be explained, according to our results, that for many, the university does not comply with some important aspects, such as offering agreements that promote student research, or the little recognition of the research interest of some students, besides, the academic community does not recognize the effort made by the university to advance in this aspect ( Mirón-Chacón *et al.*, 2020; Castro-Rodríguez, 2019).

The results indicate that providing recognition to students for their research activity is associated with performance rather than academic productivity, as previously reported in the study by Rueda-Barrios and Rodenes-Adam (2016) who found a statistically significant relationship between both variables. Universities need to promote the dissemination of research in the student body as well as to make visible the institutional effort to position the university in the research field both internally and externally, through various means that include national and international scientific journals, congresses and incentives to promote research, but that go unnoticed at the student level, as indicated by Oyarzún Maldonado *et al.* (2020). This aspect is relevant since the evidence suggests that recognition is a good practice that promotes and facilitates research management, such as the case of the universities best placed in world rankings that include incentive policies for research and those who do it ( Tomàs-Folch, Ruíz and Labao, 2015).

There are uniform criteria to study scientific research productivity, but there are gaps in the application of these principles, caused, among other aspects, by the weak dissemination of internal policies on scientific publication among students. Many times, this effort is only reflected in the fulfillment of an indicator. It is also necessary the participation of the professor who teaches research, the advisor or mentor, to accompany the deep understanding of the implications of the scientific method transmitted by these policies ( Vera and Vera, 2015).

Another important factor found in this study was related to knowledge management, a relevant aspect according to some authors since it is one of the key indicators that allows measuring the quality of universities ( Calderón, 2017). We found a concern by students since they report that there is no proper dissemination and use of the results of the research in which they participate as part of the recognition and dissemination of their activities; likewise, most do not participate in scientific networks or groups, nor do they have access to scientific journals that facilitate the publication process. This is of great importance, since according to Rueda-Barrios and Rodenes-Adam (2016) these aspects are related to scientific productivity. However, in this study, we only found a statistically significant association with the dissemination of results. This difference is probably due to the characteristics of the sample, since the authors included professionals who did research.

Even though the results state that students recognize the qualification of the professors who teach research, this result is contradictory to those identified in the externalization dimension, where they express low participation in scientific networks and the perception of scarce dissemination of research results in indexed journals. In this regard, it should be emphasized that a network of knowledge and scientific research expresses the collective multidisciplinarity of the community and institutions when establishing strategic alliances for research purposes or to develop research projects based on quality practices, strengthening scientific cooperation and efficiency in the management of human, logistic and financial resources. This is an aspect to improve in Peruvian universities if they wish to actively improve scientific knowledge management and its assumption as a new institutional challenge ( Bedoya *et al.*, 2018).

Knowledge management by researchers, whether teachers or students, demands financial, institutional, logistical resources, among others, that researchers cannot assume and that necessarily require institutional support that the university management can solve through research policies and competitive fund strategies that encourage student researchers to walk the path of research ( Ramirez, 2015; Daher, Panuncio and Hernandez, 2018). In spite of the above, there are gaps related to the development of mechanisms that influence the level of student interest and effort to publish. This raises the need to develop a research culture that contributes to consolidate and establish relationships between students, teachers and administrators, within the framework of knowledge communities, research networks and incentives for students to participate in research processes, so that the results also benefit the university ( Mendoza *et al.*, 2020).

This research finds evidence of the need to empower students as agents of change by channeling the research potential through strategies that incorporate research incentive structures for the participation and dissemination of new knowledge produced in response to a society that claims innovative solutions to the different problems that afflict the contemporary world. In this sense, the university is called to lead these research processes through an inter- and transdisciplinary approach that makes it possible to address the main scientific weaknesses of students, the ongoing training of teachers and the continuous promotion of relevant and participatory research activity of students that will result in better student scientific research productivity ( Morán-Mariños, Montesinos-Segura and Taype-Rondan, 2019).

### Limitations

The research had limitations in terms of the population included which, being composed of students from the Norbert Wiener University, may not be representative of the whole country in consideration of the socioeconomic characteristics. Likewise, the research recognizes the complexity involved in evaluating scientific production that goes beyond the classification of the journal in which it is published, the H index, the number of publications in indexed journals, language, inter-institutional or international collaboration, among others. However, the relevance of this study lies in the fact that it is one of the first at national level to evaluate the association of student scientific research productivity with variables such as institutional culture, knowledge management and technological aspects with their respective dimensions.

## Conclusions

Student performance in scientific research was associated with management commitment, the feeling of personal growth through research, the recognition provided by the university, the existence of strategic alliances, the existence of research proposals, the dissemination and use of the results obtained by the students, and the availability of technological capital. These findings are consistent with what is described in the literature, they also provide relevant information to better understand the current situation of research in university faculties. There is little student participation in research, and this is an indicator that is not properly addressed in universities to promote a scientific attitude in their students, which may have medium-term consequences in terms of academic quality and university social impact. This suggests the need to reinforce university programs or systems for the control and follow-up of student scientific activity. Finally, there is a need to conduct studies of greater scope that delve more deeply into the conditioning factors of student performance in scientific research and the future panorama at national and international level.

## Data Availability

Zenodo: Conditioning factors for the scientific productivity of undergraduate students of health sciences at a private Peruvian University. https://doi.org/10.5281/zenodo.8378295 ( Bonilla-Asalde *et al., *2023a). This project contains the following underlying data:
- Conditioning factors for the scientific productivity of undergraduate students of health sciences at a private Peruvian University.xlsx (dataset) Zenodo: Conditioning factors for the scientific productivity of undergraduate students of health sciences at a private Peruvian University: A cross-sectional analytical study (SURVEY). https://doi.org/10.5281/zenodo.10162604 ( Bonilla-Asalde *et al., *2023b). This project contains the following extended data:
- Survey (a copy of the questionnaire) Data are available under the terms of the Creative Commons Attribution 4.0 International license (CC-BY 4.0).
