# Supplementary material for: Conditioning factors for the scientific productivity of undergraduate students of health sciences at a private Peruvian University: A cross-sectional analytical study
Source: F1000Res. 2025 Jan 13;13:5. Originally published 2024 Jan 3. [Version 3] doi: 10.12688/f1000research.143021.3 (PMC11733730; doi:10.12688/f1000research.143021.3)
Supplement: Peer review history

# Reviewer report: Reviewer response for version 3

**DOI:** 10.5256/f1000research.176574.r358950

**Published:** 2025-01-22

**Authors:** Valia Luz Venegas Mejia

**Affiliations:** 1 Universidad Nacional Federico Villarreal, San Miguel, Lima Region, Peru


I have reviewed the changes and adjustments that were made to the final version of the article and I consider that they are well organized and do not affect the evaluation made initially.

I give my approval to the final version, in my role as evaluator.


Is the work clearly and accurately presented and does it cite the current literature?

Yes

If applicable, is the statistical analysis and its interpretation appropriate?

Yes

Are all the source data underlying the results available to ensure full reproducibility?

Yes

Is the study design appropriate and is the work technically sound?

Yes

Are the conclusions drawn adequately supported by the results?

Yes

Are sufficient details of methods and analysis provided to allow replication by others?

Yes

Reviewer Expertise:

NA

I confirm that I have read this submission and believe that I have an appropriate level of expertise to confirm that it is of an acceptable scientific standard.

# Reviewer report: Reviewer response for version 2

**DOI:** 10.5256/f1000research.175914.r349215

**Published:** 2024-12-26

**Authors:** Mario Valladares-Garrido

**Affiliations:** 1 Universidad Continental, Huancayo, Junin, Peru

Thank you for addressing the initial feedback so thoroughly. After reviewing the revised manuscript, I am pleased to confirm that the updates have significantly improved its quality and clarity.

 The manuscript now meets indexing standards, and I recommend its acceptance pending final editorial review.

Is the work clearly and accurately presented and does it cite the current literature?

Yes

If applicable, is the statistical analysis and its interpretation appropriate?

Yes

Are all the source data underlying the results available to ensure full reproducibility?

Yes

Is the study design appropriate and is the work technically sound?

Yes

Are the conclusions drawn adequately supported by the results?

Partly

Are sufficient details of methods and analysis provided to allow replication by others?

Partly

Reviewer Expertise:

Medical Education

I confirm that I have read this submission and believe that I have an appropriate level of expertise to confirm that it is of an acceptable scientific standard.

# Reviewer report: Reviewer response for version 1

**DOI:** 10.5256/f1000research.156636.r299561

**Published:** 2024-07-26

**Authors:** Valia Luz Venegas Mejia, José Theódulo Esquivel Grados

**Affiliations:** 1 Universidad Nacional Federico Villarreal, San Miguel, Lima Region, Peru; 2 Universidad Nacional Jose Faustino Sanchez Carrion, Huacho, Lima Region, Peru

The results provide specific information to improve the quality of the research process in universities, since the association that is revealed would allow its implementation focused on the best use of the budget, recognition and other criteria linked to the research process of the mechanisms of quality assurance of universities at the national and international level.

 Main points

 The sources are relevant and current, they allow the research to be supported. The methodological design is solid, requiring statistical techniques consistent with the findings. Meets the journal's publication criteria. The interpretations of the statistical figures and tables present the most notable data, which are supported by the discussion of the results. Tables can be easily viewed to expand information and understanding. 

 Minor points

 Although the conclusions are supported by the arguments, it would be advisable to accompany it with more relevant statistical results of the findings when the findings are described and supported.

Is the work clearly and accurately presented and does it cite the current literature?

Yes

If applicable, is the statistical analysis and its interpretation appropriate?

Yes

Are all the source data underlying the results available to ensure full reproducibility?

Yes

Is the study design appropriate and is the work technically sound?

Yes

Are the conclusions drawn adequately supported by the results?

Yes

Are sufficient details of methods and analysis provided to allow replication by others?

Yes

Reviewer Expertise:

NA

We confirm that we have read this submission and believe that we have an appropriate level of expertise to confirm that it is of an acceptable scientific standard.

# Reviewer report: Reviewer response for version 1

**DOI:** 10.5256/f1000research.156636.r282097

**Published:** 2024-07-04

**Authors:** Mario Valladares-Garrido

**Affiliations:** 1 Universidad Continental, Huancayo, Junin, Peru

**Abstract**

 The abstract is well-structured and provides a comprehensive overview of the study. However, a few improvements could enhance clarity and comprehensiveness:

1. **Background Context**: Briefly explain the significance of scientific research productivity in health sciences education.
2. **Operational Definitions**: Clarify how key variables (e.g., scientific research productivity) were defined and measured.
3. **Elaborate on Conclusion**: Offer specific recommendations for universities on how to strengthen research promotion measures based on the study's findings.

**Methods**

 The methods section is comprehensive and well-structured, with clear and appropriate methodological choices. However, a few improvements could enhance the clarity and comprehensiveness:

1. It is crucial to know the exact number of students who were excluded based on the established exclusion criteria (i.e., those who did not wish to participate in the study and those who did not properly complete the requested information).
2. It is necessary to know the parameters used to estimate the sample size of 400 students: the proportion, precision (margin of error), and confidence level. This information is essential for understanding the sample size calculation.
3. Operational Definitions: Provide more detail on how key constructs (e.g., institutional culture, knowledge management) are operationalized within the study.
4. Enhance Operational Details: Include specific examples or definitions of how independent and dependent variables were measured.

**Results**

1. The section mentions a multiple logistic regression analysis to determine associations with scientific research productivity. However, since the study design is cross-sectional and you have reported adjusted prevalence ratios (PRa), not odds ratios (OR), it would be more appropriate to refer to the analysis as a multiple regression model for estimating prevalence ratios (PR).

**Discussion**

 The discussion effectively addresses key findings and their implications but could be enhanced with more actionable insights and specific examples from other contexts to provide a clearer path forward for improving student scientific research productivity.

1. Data on Student Research Participation: The discussion highlights low research participation, but it would be useful to provide comparative context. For example, how does this participation compare with other universities or regions? This can help in understanding the relative position of the studied population.
2. Role of University Support: While the discussion mentions university support and recognition as crucial, more specifics on what kind of support (e.g., financial, mentorship, infrastructure) would be beneficial.
3. Recognition of Faculty Involvement: The role of faculty in promoting research is noted, but the discussion could expand on how faculty involvement can be enhanced. For example, how can universities incentivize faculty to mentor students in research?
4. Institutional Gaps: Identifying institutional gaps is crucial, but the discussion could be more actionable by recommending concrete steps universities can take to address these gaps, such as creating more research opportunities, providing access to scientific journals, and fostering a culture of research from early undergraduate years.
5. It would be beneficial to add a section on the implications of the findings for medical education. This section could discuss how the study's results could influence curriculum design and educational practices in health sciences faculties. Specific recommendations could focus on enhancing student engagement in scientific research, integrating research practices early in the curriculum, and strengthening institutional support and research culture to foster a scientific mindset among students.
6. The conclusions drawn are consistent with the findings, but it would be stronger with more emphasis on practical implications. How can universities implement the study’s findings? What are the next steps for research in this area?

**Limitations:**

1. Potential Information Bias: The study should acknowledge the possibility of information bias due to unmeasured variables that could act as confounders.
2. Selection Bias: The study's focus on students from Norbert Wiener University raises concerns about selection bias. The lack of representativeness of the broader student population may limit the generalizability of the findings. It's essential to explicitly state how this bias might have influenced the results and the extent to which the findings can be applied to other universities or contexts.
3. Participation Bias: The potential for participation bias should be addressed, particularly if students who participated in the study differ systematically from those who did not.

Is the work clearly and accurately presented and does it cite the current literature?

Yes

If applicable, is the statistical analysis and its interpretation appropriate?

Yes

Are all the source data underlying the results available to ensure full reproducibility?

Yes

Is the study design appropriate and is the work technically sound?

Yes

Are the conclusions drawn adequately supported by the results?

Partly

Are sufficient details of methods and analysis provided to allow replication by others?

Partly

Reviewer Expertise:

Medical Education

I confirm that I have read this submission and believe that I have an appropriate level of expertise to confirm that it is of an acceptable scientific standard, however I have significant reservations, as outlined above.
